# Quality of Life, Social Support, Acceptance of Illness, and Self-Efficacy among Pregnant Women with Hyperglycemia

**DOI:** 10.3390/ijerph16203941

**Published:** 2019-10-16

**Authors:** Grażyna Iwanowicz-Palus, Marta Zarajczyk, Beata Pięta, Agnieszka Bień

**Affiliations:** 1 Chair and Department of Development in Midwifery, Faculty of Health Sciences, Medical University of Lublin, 4-6 Staszica St., 20-081 Lublin, Poland; spupalus@gmail.com (G.I.-P.); agnesmbien@gmail.com (A.B.); 2 Department of Maternal and Child Health, Department of Midwifery, Poznan University of Medical Science, 41 Jackowskiego St., 60-533 Poznan, Poland; biataid@wp.pl

**Keywords:** pregnancy, diabetes mellitus, hyperglycemia, quality of life, social support, acceptance of illness, generalized self-efficacy

## Abstract

Carbohydrate metabolism disorders resulting in hyperglycemia are among the most common metabolic complications of pregnancy. According to 2017 data from the International Diabetes Federation (IDF), 16.2% of pregnancies are complicated with hyperglycemia, of which gestational diabetes mellitus (GDM) accounts for 86.4% of cases. Carbohydrate metabolism disorders developing during pregnancy require the patient to change her lifestyle or, in some cases, to undergo pharmaceutical treatment, which may affect various aspects of the patient’s life, including her perceived quality of life (QoL). The purpose of the present study was to evaluate levels of QoL, social support, acceptance of illness, and self-efficacy among pregnant patients with hyperglycemia. The study was performed between July 2016 and September 2017 in a group of hyperglycemic pregnant women. The following instruments were used: the World Health Organization Quality of Life—BREF (WHOQOL-BREF), the Berlin Social Support Scales (BSSS), the Acceptance of Illness Scale (AIS), the Generalized Self-Efficacy Scale (GSES) and a standardized interview questionnaire. Participants rated their overall QoL (3.64 points) higher than their overall perceived health (3.43). In terms of social support, the highest scores were obtained in terms of actually received support (3.53) and perceived available instrumental support (3.52), while the lowest in terms of support seeking (2.99) and the need for support (2.95). The mean acceptance of illness score among the hyperglycemic pregnant women that were studied was 31.37, and the mean generalized self-efficacy score was 31.58. Participants’ reported QoL in the various WHOQOL-BREF domains was associated with specific social support scales, acceptance of illness, and generalized self-efficacy.

## 1. Introduction

In the context of a global obesity and diabetes epidemic, research demonstrates a constant upward trend in the number of women with carbohydrate metabolism disorders during pregnancy. According to 2017 data from the International Diabetes Federation (IDF), 16.2% of pregnancies are complicated with hyperglycemia, of which gestational diabetes mellitus (GDM) accounts for 86.4% of cases [1,2]. Other sources report that hyperglycemia occurs as a complication of pregnancy in 1%–22% of cases worldwide. This large variation in estimates of the incidence of hyperglycemia in pregnant women is due to the heterogeneous protocols for diagnosing and classifying the disorder used in various regions of the world [3,4,5,6,7,8,9,10,11,12,13,14].

Regardless of the form of hyperglycemia that occurs during the pregnancy, it entails a risk of complications both for the pregnant woman and for her child and affects their future health. The occurrence of carbohydrate metabolism disorders during pregnancy requires the patient to change her lifestyle or, in some cases, to undergo pharmaceutical treatment, which may affect the patient’s perceived quality of life (QoL) [2,15,16].

In 1993, a group of experts from the WHO Quality of Life Group (WHOQOL) published a definition of QoL that provided a novel outlook on health and disease, in response to the increasing interest in the notion of QoL within medical science [17,18]. Under this definition, QoL is determined by an individual’s perception of their life situation in the context of their culture, values, goals, interests, expectations, and standards. Thus, the definition puts the focus on a subjective and multi-dimensional understanding of QoL [17,18,19]. 

In chronic illness, the patient’s psychological condition, which depends on their family relations and available social support, is of paramount importance to care. It affects, among other factors, the extent to which the patient adapts to the difficulties and lifestyle changes associated with the disease. In practice, tracking acceptance of illness, generalized self-efficacy, and available and perceived social support in patients may increase the effectiveness of care for these patients, particularly in cases of chronic illness [20,21,22].

The purpose of the present study was to evaluate levels of QoL, social support, acceptance of illness, and self-efficacy among pregnant patients with hyperglycemia.

## 2. Materials and Methods

### 2.1. Subjects

The study was performed between July 2016 and September 2017 in Lublin Province, Poland, in a group of 339 hyperglycemic pregnant women. The inclusion criteria were as follows: age above 18 years; consent to participate in the study; time from hyperglycemia diagnosis exceeding 5 weeks; using health care in Poland throughout the pregnancy; and hyperglycemia diagnosed before or during the current pregnancy as per the current Polish Diabetes Association guidelines:

→ gestational diabetes mellitus (GDM) is diagnosed when at least one of the following criteria is met in a 75g Oral Glucose Tolerance Test (OGTT): fasting glucose 92–125 mg/dL (5.1–6.9 mmol/L), glucose level at 60 minutes ≥180 mg/dL (10 mmol/L) or glucose level at 2 hours 153–199 mg/dL (8.5–11 mmol/L);
→ diabetes in pregnancy (DIP) is diagnosed when at least one of the following criteria is met: fasting glucose over 126 mg/dL (7 mmol/L), glucose level at 2 hours in 75g OGTT ≥200 mg/dL (11.1 mmol/L), or casual glucose level exceeding 200 mg/dL (11.1 mmol/L) with clinical hyperglycemic symptoms [23].

Diagnosis of other pregnancy complications, such as hypertension, imminent premature birth, thyroid disease, liver disease, etc., which could affect the pregnant patients’ perception of their quality of life and social support, was an exclusion criterion for study group.

The study was approved by the Medical University of Lublin Bioethics Committee (decision no. KE-0254/160/2016). Permission was also obtained from each health care institution where the study was performed. Respondents were informed that participation was anonymous, and freely provided their consent to participate. Before the start of the study, they were also informed that any findings would only be used for research purposes.

### 2.2. Assessments

→ The study was performed using the diagnostic survey method with questionnaires. The following instruments were used: the World Health Organization Quality of Life—BREF (WHOQOL-BREF), the Berlin Social Support Scales (BSSS), the Acceptance of Illness Scale (AIS), the Generalized Self-Efficacy Scale (GSES) and a standardized interview questionnaire designed to record the participants’ characteristics.
→ The World Health Organization Quality of Life—BREF (WHOQOL-BREF) test, which allows for evaluating QoL both in healthy and ill individuals in clinical practice, was developed on the basis of the WHOQOL-100 questionnaire. It has been adapted for use in Polish settings by L. Wołowicka and K. Jaracz. It includes 26 items referring to situations experienced by the patient in the previous 4 weeks. Its first 2 items involve the patient’s subjective view of their overall QoL and overall health. The remaining items allow for an assessment of 4 QoL domains: physical (somatic), psychological, social, and environmental. Responses are provided using a 5-item scale for a score of 1 to 5. In each domain, the score is positive, i.e., higher scores reflect a better QoL as perceived by the respondent. Internal consistency as measured by Cronbach’s α for each of the scale’s dimensions ranges between 0.54 and 0.91. Cronbach’s α for the entire scale is 0.92 in healthy individuals, and 0.95 in ill individuals [17,19].

The Berlin Social Support Scales (BSSS) by R. Schwarzer and U. Schutz, adapted into Polish by A. Łuszczyńska and M. Kowalska, comprise 6 subscales. In the present study, the following subscales were used: I—perceived available support, II—need for support, III—support seeking, V—actually received support, VI—protective buffering (subscale IV—actually provided support was not used). Items are scored on a scale of 1 to 4, where 1 indicates that the respondent considers the statement completely false, and 4 indicates they consider the statement completely true. Higher scores indicate more social support. Cronbach’s α for the questionnaire is 0.80 [24].

The Acceptance of Illness Scale (AIS) was developed by B.J. Felton et al. in 1984, and adapted for use in Polish settings by Z. Juczyński. The scale may be used for any illness. It measures the level of illness acceptance in adult patients. The AIS comprises 8 items describing the negative consequences of poor health, rated using a 5-point scale ranging from 1—completely agree, to 5—completely disagree. The final acceptance of illness score is a sum of points from all items, between 8 and 40 points. The higher the score, the more a patient accepts their illness, which results in better adaptation to the limitations resulting from the condition and less psychological discomfort. Cronbach’s α for the Polish version is 0.85, indicating a reliability similar to that of the original, which has a Cronbach’s α of 0.82 [25].

The Generalized Self-Efficacy Scale (GSES) developed by R. Schwarzer and M. Jerusalem was adapted into Polish by Z. Juczyński. The questionnaire may be used in healthy or ill adult patients, and comprises 10 items. For each item, 1 of 4 responses must be selected: 1—not true at all, 2—hardly true, 3—moderately true, 4—exactly true. The scale measures an individual’s general perception of their efficacy in dealing with obstacles and difficult situations on a daily basis. The total score, ranging between 10 and 40 points, is an indicator of generalized self-efficacy, with higher scores denoting a stronger sense of self-efficacy. Scores should be interpreted using sten ranges, with scores of sten 1–4 considered low, and sten 7–10 considered high. Cronbach’s α for the questionnaire is 0.85 [26].

### 2.3. Statistical Analyses

Statistical analysis of the collected material was performed using the IBM SPSS Statistics (v. 21) software. Quantitative variables were described using mean (M), standard deviation (SD) and median (Me). For qualitative variables, numbers and percentages in each category were given. The following tests were used when the assumptions for parametric tests had been met (quantitative measurement): Student’s t-test (t) for independent groups, which verifies whether the means of the variable analyzed are equal in 2 populations, and one-way ANOVA (F) for independent groups, which verifies whether the means of the variable analyzed are equal in several populations. In the case of differences revealed by the ANOVA results, post-hoc tests were performed: Tukey’s test (equal variances) and the Games-Howell test (unequal variances).

A series of regression analyses were also performed, with all explanatory variables introduced in the model. The results were interpreted by comparison of the β coefficient (β), in accordance with the correlation strength and direction for each predictor. The variables that were explained included the physical, psychological, social, and environmental QoL domains (WHOQOL-BREF). Explanatory variables were: GSES score, AIS score, and BSSS subscales: perceived emotional support, perceived instrumental support, need for support, support seeking, and actually received support. 

The choice of variables that were explained was preceded with correlation analysis and observation of the scatter diagram (outlier analysis). The following assumptions were also verified: correlation of residuals: the result of the Durbin–Watson statistic was around 2; correlation of predictors: VIF did not exceed 10, and the tolerance was not lower than 0.2. In the case of two predictors, skewness and kurtosis were high, thus logarithmic transformation was performed on raw data. The study used a significance threshold of *p* < 0.05.

## 3. Results

Among the pregnant women with hyperglycemia in the study group, most were: aged 26–30 (31.9%), married (88.5%), living in province capitals (39.5%), holders of a master’s degree (43.1%), professionally active (61.1%), living in good conditions (53.1%), pregnant for the first time (37.8%), diagnosed with hyperglycemia between weeks 24 and 28 of the current pregnancy (53.4%) and treated with a diabetic diet and exercise (64.9%)—Table 1.

Table 2 shows the QoL scores for the patients studied. Participants rated their overall QoL (3.64 points) higher than their overall perceived health (3.43). Of the remaining domains, the highest scores were obtained in the social domain (15.21), followed by the psychological (14.92) and environmental domains (14.88). Scores in the physical domain were the lowest (12.60).

Table 3 shows an analysis of social support in the study group. The highest scores were obtained in terms of actually received support (3.53) and perceived available instrumental support (3.52), while the lowest were in terms of support seeking (2.99) and need for support (2.95).

Table 4 presents an analysis of correlations between QoL and socio-demographic variables among pregnant women with hyperglycemia. If the variables correlated with each another, post-hoc tests were performed to identify those variable pairs which displayed statistically significant correlations. The statistical analysis did not demonstrate statistically significant correlations between the age of the women studied and individual QoL domains (*p* > 0.05). The analysis of QoL and marital status revealed that married women experienced higher QoL in the psychological and environmental domains than single women (*p* < 0.05). The highest levels of overall QoL, overall perceived health and QoL in the environmental domain were observed in pregnant women living in rural areas, the lowest were observed in pregnant women living in cities other than province capitals (*p* < 0.05). The highest scores in the psychological and environmental domains were obtained by women with higher education, and the lowest by respondents with primary or vocational education. As for the social domain, the highest scores were also observed in women with higher education, and the lowest in those with vocational education. The lowest QoL in the social and environmental domains was demonstrated by professionally active pregnant women with hyperglycemia. The highest scores in each of the QoL domains were obtained by respondents who reported their living conditions as very good, and the lowest by women who reported their living conditions as average/poor (*p* < 0.001). The highest QoL in the psychological and social domains was experienced by women who were pregnant for the first time, and the lowest by those who were pregnant three or more times. The overall QoL and QoL in the psychological domain were higher in women treated with a diabetic diet and exercise than those treated with a diabetic diet and insulin (*p* < 0.05)—Table 4. 

The next step involved the analysis of correlations between social support and socio-demographic variables among the pregnant women studied. If the variables correlated with each another, post-hoc tests were performed to identify those variable pairs which displayed statistically significant correlations.

There was no statistically significant correlation between social support and the age of the pregnant women studied (*p* > 0.05). 

On the other hand, marital status correlated with perceived emotional and instrumental support. Higher scores were observed in the group of married women (*p* < 0.05). A statistically significant correlation was also noted between the place of residence and social support. In the case of perceived emotional and instrumental support, the highest scores were observed in women living in a province capital, and the lowest in those living in cities other than province capitals. On the other hand, women living in rural areas obtained the highest scores in terms of the need for support, support seeking, and actually received support. These areas were rated lowest by women living in cities other than province capitals. 

Correlations were also found between perceived emotional and instrumental support and actually received support, and the education of the pregnant women studied (*p* < 0.001). The highest scores in this respect were observed in holders of a master’s degree. The lowest scores in perceived instrumental support and actually received support scales were obtained by women with primary or vocational education, and in the emotional support scale by women with vocational education. 

The analysis demonstrated statistically significant differences between professional activity of the women studied and perceived emotional and instrumental support, support seeking and actually received support. Professionally active respondents obtained higher scores in the social support scales than those inactive (*p* < 0.05). Statistically significant correlations were also observed between self-reported living conditions and all social support scales. Respondents who reported their living conditions as average/poor obtained the lowest scores in each of the scales (*p* < 0.05). 

The analysis showed a correlation between social support and the number of pregnancies among pregnant women with hyperglycemia. The level of perceived emotional support, perceived instrumental support and support seeking decreased together with subsequent pregnancies (*p* < 0.05). Higher need for social support was found in women treated with a diabetic diet and exercise (*p* < 0.05) (Table 5).

Mean acceptance of illness score in the study group was 31.37, and the mean generalized self-efficacy score was 31.58, or sten 7, indicating a strong sense of self-efficacy among the women—Table 6.

Table 7 shows the results of regression analysis for the specific WHOQOL-BREF domains, social support, acceptance of illness, and generalized self-efficacy. In the case of the physical QoL domain, the proposed regression model had a good fit to the data (*F* = 14.380; *p* < 0.001). The model that was tested accounted for 23% of variance in the variable. QoL in the domain had a weak positive correlation with acceptance of illness (*β* = 0.270, *p* < 0.001) and self-efficacy (*β* = 0.147, *p* = 0.008). Higher QoL in the physical domain was associated with higher levels of acceptance and self-efficacy in the hyperglycemic pregnant women.

In the case of the psychological QoL domain, the proposed regression model also had a good fit to the data (*F* = 47.962; *p* < 0.001). It accounted for 50% of variance in the psychological QoL score. QoL in the domain had a moderately strong positive correlation with acceptance of illness (*β* = 0.415, *p* < 0.001), and weak positive correlations with perceived available instrumental support (*β* = 0.232, *p* < 0.001) and self-efficacy (*β* = 0.171, *p* < 0.001). 

Next, the social domain was analyzed. The proposed regression model had a good fit to the data (*F* = 25.672; *p* < 0.001). QoL in the social domain had weak positive correlations with perceived available emotional support (*β* = 0.175, *p* = 0.020), actually received support (*β* = 0.162, *p* = 0.006), acceptance of illness (*β* = 0.215, *p* = 0.000), and generalized self-efficacy (*β* = 0.141, *p* = 0.005). The model accounted for 35% of variance in the social QoL score. Women who scored higher in the social QoL domain also scored higher in terms of perceived available emotional support, actually received support, acceptance of illness, and generalized self-efficacy.

Moreover, in the case of the environmental QoL domain, the proposed regression model also had a good fit to the data (*F* = 38.653; *p* < 0.001). QoL in the environmental domain had weak positive correlations with perceived available emotional support (*β* = 0.203, *p* = 0.003), perceived available instrumental support (*β* = 0.164, *p* = 0.017), actually received support (*β* = 0.194, *p* < 0.001), acceptance of illness (*β* = 0.214, *p* < 0.001), and generalized self-efficacy (*β* = 0.164, *p* < 0.001). The model accounted for 45% of variance in the environmental QoL score. Hyperglycemic pregnant women who scored higher in the environmental QoL domain also scored higher in terms of perceived available emotional and instrumental support, actually received support, acceptance of illness, and generalized self-efficacy.

## 4. Discussion

A diagnosis of hyperglycemia requires the pregnant patient to change her lifestyle, which may affect her wellbeing, or cause a sense of losing control over her own body, thus changing her perceived health and QoL [15,27,28]. Worldwide, numerous studies have been published on the adverse impact of type 1 and 2 diabetes mellitus on self-reported QoL in children and adults [29,30,31]. Data on the impact of hyperglycemia diagnosis on QoL and perceived health in women under the special circumstances associated with pregnancy still remain scarce [15,28,32,33].

QoL scores in the specific WHOQOL-BREF domains obtained in the present study are to some extent consistent with those reported by other researchers. As was the case with the present findings, Bień et al. (2016) reported that pregnant women with hyperglycemia scored higher for overall QoL than for overall perceived health [15]. This finding has also been corroborated by other authors, who found an adverse impact of GDM diagnosis on women’s self-reported health [27,28]. In a study by Kalka (2016), in a group of women with type 2 diabetes aged 45–55, overall quality of life was scored higher than overall perceived health [29].

For specific WHOQOL-BREF domains, among the pregnant women with hyperglycemia studied here, the highest scores were found in the social domain, in line with other reports on QoL in pregnant women, pregnant women with diabetes, and postpartum women after a pregnancy complicated by hyperglycemia [15,34,35,36]. 

In the present study, the lowest scores were obtained in the physical QoL domain. The same finding was also reported by Marquesim et al. (2016) in a study on pregnant women with diabetes or mild hyperglycemia [35]. In studies by Bień et al. (2016) and Mautner et al. (2009), the lowest QoL scores were found in the psychological domain [15,36]. The psychological domain of the WHOQOL-BREF questionnaire was also rated lowest in the study by Gholami et al. (2013) on a group of type 2 diabetes patients [34].

The present study, in line with reports by other authors, demonstrates that women with hyperglycemia, during the pregnancy or otherwise, are eager to maintain social relationships, but experience multiple restrictions in the performance of their social roles, as their chronic illness interferes with a number of aspects of human functioning, both in the physical and in the psychological domain [15,36]. 

There was a correlation between participants’ reported QoL and their marital status. Married pregnant women with hyperglycemia rated their QoL in the psychological and environmental domains higher than single women. A study by Ramirez (2011) conducted among pregnant women in Columbia yielded results that are consistent with our findings [37]. 

Higher scores obtained by pregnant women with hyperglycemia in terms of reported living conditions correlated with higher overall QoL, perceived health and QoL in the other domains. In a study by Bień et al. (2016), such a correlation was observed in the case of physical, psychological and environmental domains [15]. Research by Sekhar et al. (2018) conducted among women with gestational diabetes [28] and by Ramirez (2011) conducted among pregnant women [37] demonstrated that the participants’ higher economic status translated into higher QoL.

In our study, the type of treatment used affected perceived QoL in the pregnant women. The highest scores in the overall QoL and the psychological domain were observed in women treated with a diabetic diet and exercise, and the lowest in those treated with insulin. These findings are consistent with the results obtained by Bień et al. (2016), Sekhar et al. (2018) and Latif et al. (2013), who demonstrated that the use of insulin substantially decreased the level of reported QoL in pregnant women with hyperglycemia [15,28,33]. On the other hand, a study by Trutnovsky et al. (2012) conducted among women with gestational diabetes produced results that are only partially in line with ours [38]. The authors indicate that lower QoL is observed only at the beginning of insulin treatment. The fear of injection and inept administration of insulin, which affects perceived QoL, subsides as a result of adequate education, thus increasing the level of reported QoL [38]. Studies by Dalfrá et al. (2012) and Rwegerer et al. (2018), on the other hand, showed that insulin treatment did not cause QoL decrease in pregnant and non-pregnant women with diabetes. According to the authors, this stemmed from a better possibility to control glycaemia as compared to treatment with diet alone [31,32].

Pregnancy is a special time in a woman’s life. For some, it is a joyful time of expectation, while for others it is a period of new psychological difficulties that often come with the changes occurring in the body and its functions. Complications make pregnancy even more difficult for the expectant mother. One factor that affects a woman’s well-being during a normal pregnancy, and even more when pregnancy complications occur, is social support [28,39,40,41]. Research to date has demonstrated a positive impact of social support on a number of aspects of physical and psychological health, as well as on the ability to cope with difficulties, thus improving the overall well-being of an individual faced with new and often stressful situations in life [28,39,40,42]. However, in the literature on the subject of social support, studies on women with hyperglycemia during the pregnancy remain scarce [28].

In the present study, pregnant women with hyperglycemia scored higher in terms of actually received support than in terms of need for support and support seeking. Similar findings were reported by Iranzad et al. (2014), who found a high level of social support in the group of pregnant women they studied [43]. 

One of the socio-demographic factors that differentiated the level of social support among the women studied was marital status. In our study, it affected the perceived available support, both emotional and instrumental. Married women obtained higher scores in the respective two BSSS subscales. A higher level of social support among married women was also observed by Ramkisson et al. (2017) [42]. Women who obtained high scores in terms of social support coped better with self-observation, self-care and treatment during disease, and presented a lower level of stress [42].

The highest perceived emotional and instrumental support was reported by women living in a province capital. Higher perceived availability of support in these scales was most likely related to better infrastructure in province capitals, which translates into higher accessibility of specialists. On the other hand, the highest scores in terms of the need for support, support seeking and actually received support were found in women living in rural areas. These findings correspond with the results obtained by Edmonds et al. (2011) and Ahmed et al. (2017), who believe that such scores are determined by the nature of village dwellers [39,44]. These people are more willing to offer their support, do not feel embarrassed to ask for help and show more appreciation of the support received from others [39]. 

Our study showed that women with higher education scored highest in the perceived available emotional, instrumental and actually received support scales. These results are in line with those reported by Azimi et al. (2018) and Abdollahpour et al. (2015), who studied pregnant women [45,46]. 

Our analysis showed that women who obtained higher scores in terms of their living conditions reported higher social support in all social support scales. Other researchers are of a similar opinion: Mirabzadeh et al. (2013) demonstrated higher support in the case of higher socioeconomic status [47], and Azimi et al. (2018) reported less extensive social support networks among primiparas with a low income [46]. Ahmed et al. (2017) demonstrated a higher level of social support in women earning average incomes [39]. In contrast, Shishehgar et al. (2016) and Abdollahpour et al. (2015) did not find any correlation between living conditions and social support among pregnant women [45,48].

In our study, the highest scores in the perceived available emotional and instrumental support, and support seeking scales were observed in women who were pregnant for the first time. Gebuza et al. (2016) reported similar results assessing social support among pregnant women [40]. 

The need for dietary changes following the diagnosis of hyperglycemia during pregnancy is one of the most difficult challenges accompanying the illness. Ramkisson et al. (2017) found that nearly half of the diabetic respondents had the need for help and support with regard to meal preparation [42]. Our results were similar in this respect. The greatest need for support was presented by pregnant women who were treated with diet and exercise only. 

The present study demonstrates a correlation between QoL and social support in pregnant women with hyperglycemia. Higher scores in specific WHOQOL-BREF domains were associated with more social support in BSSS subscales. Better QoL in the psychological WHOQOL-BREF domain in the hyperglycemic pregnant women who were studied was correlated with greater perceived available instrumental support; in the social domain, with perceived available emotional support and actually received support; and in the environmental domain, with greater perceived available emotional and instrumental support, and actually received social support. 

Similar correlations were reported in a study by Sekhar et al. (2018), where intense treatment and a low level of social support were associated with increased stress, resulting in considerably poorer self-reported QoL in pregnant women with hyperglycemia [28]. Ramkisson et al. (2017) reported similar findings in a group of adult patients with type 2 diabetes [42]. Likewise, Mirabzadeh et al. (2013) reported that support provided to pregnant women contributes to their better psychological health, as well as to fewer perinatal complications, thus resulting in better perceived health and QoL in the patients [47]. In a group of type 1 and 2 diabetes patients studied by Peimani et al. (2018), more social support also correlated with higher QoL [49].

Acceptance of illness is both an indicator of the patient’s adaptation to their illness and a prognostic factor for the patient’s QoL. The way an individual perceives their illness and the resulting restrictions affects their attitudes, e.g., towards treatment. Patients with a high level of illness acceptance adapt better to the necessary lifestyle changes associated with an illness, especially a chronic one [25,50].

In the present study, the mean acceptance score among the hyperglycemic pregnant women was 31.37. Comparison with reports by other authors demonstrates that the mean acceptance level was higher in hyperglycemic pregnant women than in diabetic patients [25,30,51]. 

The present study also showed an association between acceptance of illness and QoL in pregnant women with hyperglycemia. Higher scores in each QoL domain were correlated with higher acceptance scores. This was consistent with findings reported by Bień et al. (2016) [15]. A similar report was published by Schmitt et al. (2018), who showed that a low level of illness acceptance resulted in poorer self-reported QoL in diabetic patients [52]. 

Another psychological resource that affects patients’ health-related behaviors is generalized self-efficacy. The term refers to a belief in one’s capability of changing one’s behavior so as to cope with problems and difficulties experienced in life. Self-efficacy is also a major determinant of behaviors aimed at improving or preserving one’s health. Individuals with a high level of self-efficacy also tend to set higher objectives for themselves and pursue these objectives more actively, as well as to have better self-monitoring and self-care skills in a situation of chronic illness [53,54]. A literature review yielded very few studies on the impact of self-efficacy on the life of patients with diabetes, and even fewer specifically focusing on women with gestational diabetes [49,53,54]. 

In the present study, the mean score in the GSES questionnaire was within sten 7 (31.58 points), indicating a high level of generalized self-efficacy in the women studied. A similar finding was reported by Linden et al. (2016), who analyzed self-efficacy in women with type 1 diabetes in early pregnancy [55]. 

The present analyses demonstrated that QoL had an impact on the generalized self-efficacy of pregnant women with hyperglycemia. Those who rated their physical, psychological, social, and environmental QoL higher also had higher scores in the generalized self-efficacy scale. This is consistent with reports by Linden et al. (2016) and Weber-Rajek et al. (2014), who demonstrated an association between high generalized self-efficacy and better health-related QoL reported by women with type 1 diabetes in early pregnancy, as well as better QoL reported by patients after an ischemic stroke [55,56]. A similar association was reported by Walker (2014) in a group of diabetic patients [57].

Our study on pregnant women’s QoL adds to the knowledge on the potential psychological effects of somatic health in pregnant women with hyperglycemia. Appropriate management by the therapeutic team, whose members know the individual needs of patients, may contribute to optimized obstetric care for women with hyperglycemia during pregnancy.

## 5. Conclusions

Pregnant women with hyperglycemia rate their overall QoL higher than their overall perceived health. In terms of specific domains, their QoL is the highest in the social domain, and the lowest in the physical domain. The highest levels of social support were found in terms of actually received support and perceived available instrumental support, while the lowest in terms of support seeking and need for support. 

Socio-demographic factors such as marital status, place of residence, education, professional activity, number of pregnancies and treatment used determine the level of QoL and social support among pregnant women with hyperglycemia.

Pregnant women with hyperglycemia have high levels of illness acceptance and generalized self-efficacy. Better QoL in the psychological WHOQOL-BREF domain in the hyperglycemic pregnant women studied was correlated with greater perceived available instrumental support; in the social domain, with perceived available emotional support and actually received support; and in the environmental domain, with greater perceived available emotional and instrumental support, and actually received social support. Higher QoL scores obtained by hyperglycemic pregnant women in specific WHOQOL-BREF domains (physical, psychological, social, and environmental) are associated with higher levels of illness acceptance and generalized self-efficacy. 

## Figures and Tables

**Table 1:** Socio-demographic characteristics of the hyperglycemic pregnant women studied.

Socio-Demographic Data		*n*	%
		339	100
Age	18–25 years	60	17.7
	26–30 years	108	31.9
	31–35 years	99	29.2
	36–40 years	55	16.2
	>40 years	17	5.0
Marital status	single	39	11.5
	married	300	88.5
Residence	urban–province capital	134	39.5
	urban–other	106	31.3
	rural	99	29.2
Education	primary or vocational	120	35.4
	bachelor’s degree	73	21.5
	master’s degree	146	43.1
Professional activity	professionally active	207	61.1
	professionally inactive	132	38.9
Self-reported living conditions	very good	101	29.8
	good	180	53.1
	average/poor	58	17.1
Number of pregnancies	first pregnancy	128	37.8
	second pregnancy	117	34.5
	third or subsequent pregnancy	94	27.7
Time of glucose metabolism disorder diagnosis	before the current pregnancy	35	10.3
	at first visit at the beginning of pregnancy (before week 12)	50	14.3
	between weeks 12 and 23	61	18.0
	at screening between weeks 24 and 28	181	53.4
	after week 28	12	3.5
Treatment	diabetic diet + exercise	220	64.9
	diabetic diet + insulin	119	35.1

**Table 2:** World Health Organization Quality of Life—BREF (WHOQOL-BREF) scores in the hyperglycemic pregnant women studied.

WHOQOL-BREF Domains	M	SD	Me
General Quality of Life	3.64	0.88	4.00
General Health	3.43	0.83	4.00
Physical Health	12.60	1.71	12.57
Psychological	14.92	2.36	15.33
Social Relationships	15.21	2.52	16.00
Environment	14.88	2.35	15.00

M—mean; SD—standard deviation; Me—median.

**Table 3:** Berlin Social Support Scales (BSSS) in the hyperglycemic pregnant women studied.

BSSS Scales	M	SD	Me
Perceived Emotional Support	3.39	0.51	3.50
Perceived Instrumental Support	3.52	0.58	3.75
Need for Support	2.95	0.53	3.00
Support Seeking	2.99	0.66	3.00
Actually Received Support	3.53	0.53	3.80

M—mean; SD—standard deviation; Me—median.

**Table 4:** Analysis of correlations between quality of life (WHOQOL-BREF) and socio-demographic variables among pregnant women with hyperglycemia.

Socio-Demographic Data		General Quality of Life		General Health		Physical Health		Psychological		Social Relationships		Environment	
		M	*p* (Post Hoc)	M	*p* (Post Hoc)	M	*p* (Post Hoc)	M	*p* (Post Hoc)	M	*p* (Post Hoc)	M	*p* (Post Hoc)
Age	18–25 years (1)	3.60	0.365 (-)	3.38	0.588 (-)	12.58	0.605 (-)	15.07	0.827 (-)	15.71	0.270 (-)	14.71	0.425 (-)
	26–30 years (2)	3.59		3.37		12.50		14.73		14.88		14.63	
	31–35 years (3)	3.77		3.49		12.55		15.09		15.33		15.23	
	36–40 years (4)	3.64		3.55		12.95		14.82		14.98		14.96	
	more than 40 years (5)	3.35		3.29		12.54		14.94		15.53		14.71	
Marital status	married	3.67	0.154	3.46	0.178	12.65	0.179	15.03	0.043	15.27	0.148	15.00	0.023
	single	3.44		3.23		12.25		14.10		14.74		13.92	
Residence	urban–province capital (1)	3.63	0.042	3.43	0.042	12.63	0.508 (-)	14.93	0.174 (-)	15.42	0.074 (-)	15.04	0.042
	urban–other (2)	3.50	1–2	3.29	1–2	12.45		14.62		14.74		14.27	1–2
	Rural (3)	3.81	2–3 *	3.59	2–3 *	12.73		15.24		15.41		15.31	2–3 *
			1–3		1–3								1–3
Education	primary or vocational (1)	3.58	0.423 (-)	3.35	0.153 (-)	12.38	0.055 (-)	14.54	0.020	14.97	0.041	14.08	<0.001
	bachelor’s degree (2)	3.59		3.37		12.45		14.74	1–2	14.81	1–2	14.62	1–2
	master’s degree (3)	3.71		3.53		12.86		15.32	2–3	15.60	2–3 *	15.66	2–3 *
									1–3 *		1–3		1–3 *
Professional activity	professionally active	3.65	0.753	3.45	0.572	12.71	0.148	15.08	0.118	15.42	0.050	15.21	0.001
	professionally inactive	3.62		3.40		12.43		14.67		14.87		14.36	
Self-reported living conditions	very good (1)	3.93	<0.001	3.68	<0.001	12.98	0.001	16.09	<0.001	15.96	<0.001	16.24	<0.001
	good (2)	3.63	1–2 *	3.44	1–2 *	12.60	1–2	14.63	1–2 *	15.27	1–2	14.69	1–2 *
	average/poor (3)	3.16	2–3 *	2.98	2–3 *	11.96	2–3 *	13.80	2–3 *	13.68	2–3 *	13.10	2–3 *
			1–3 *		1–3 *		1–3 *		1–3 *		1–3 *		1–3 *
Number of pregnancies	first pregnancy (1)	3.73	0.327 (-)	3.52	0.294 (-)	12.78	0.293 (-)	15.41	0.008	15.72	0.013	15.24	0.073 (-)
	second pregnancy (2)	3.62		3.39		12.44		14.77	1–2	14.94	1–2 *	14.74	
	third or subsequent pregnancy (3)	3.55		3.36		12.55		14.45	2–3	14.84	2–3	14.55	
									1–3 *		1–3 *		
Treatment	diabetic diet + exercise	3.71	0.049	3.46	0.369	12.64	0.562	15.12	0.032	15.25	0.671	15.03	0.121
	diabetic diet + insulin	3.51		3.38		12.53		14.55		15.13		14.61	

* adjusted significance.

**Table 5:** Analysis of correlations between social support scores (BSSS) and socio-demographic variables among pregnant women with hyperglycemia.

Socio-Demographic Data		Perceived Emotional Support		Perceived Instrumental Support		Need for Support		Support Seeking		Actually Received Support	
		M	*p* (Post Hoc)	M	*p* (Post Hoc)	M	*p* (Post Hoc)	M	*p* (Post Hoc)	M	*p* (Post Hoc)
Age	18–25 years (1)	3.33	0.497 (-)	3.46	0.063 (-)	2.98	0.312 (-)	3.08	0.405 (-)	3.43	0.165 (-)
	26–30 years (2)	3.36		3.46		2.92		2.97		3.52	
	31–35 years (3)	3.46		3.66		3.03		3.03		3.64	
	36–40 years (4)	3.40		3.51		2.86		2.89		3.50	
	more than 40 years (5)	3.43		3.34		2.88		2.82		3.45	
Marital status	married	3.43	0.009	3.56	0.001	2.95	0.648	2.99	0.656	3.57	0.052
	single	3.15		3.24		2.97		2.95		3.26	
Residence	urban–province capital (1)	3.50	<0.001	3.61	0.005	2.96	0.001	3.05	0.012	3.60	<0.001
	urban–other (2)	3.22	1–2 *	3.37	1–2 *	2.81	1–2 *	2.83	1–2	3.35	1–2 *
	rural (3)	3.44	2–3 *	3.56	2–3	3.08	2–3	3.07	2–3	3.64	2–3 *
			1–3		1–3		1–3		1–3 *		1–3
Education	primary or vocational (1)	3.29	<0.001	3.38	<0.001	2.98	0.310 (-)	2.96	0.075 (-)	3.41	<0.001
	bachelor’s degree (2)	3.27	1–2	3.39	1–2	2.87		2.87		3.42	1–2
	master’s degree (3)	3.54	2–3 *	3.70	2–3 *	2.97		3.07		3.69	2–3 *
			1–3 *		1–3 *						1–3 *
Professional activity	professionally active	3.45	0.010	3.61	0.001	2.99	0.059	3.08	0.001	3.59	0.011
	professionally inactive	3.30		3.39		2.88		2.85		3.44	
Self-reported living conditions	very good (1)	3.58	<0.001	3.77	<0.001	2.99	0.005	3.06	0.001	3.72	<0.001
	good (2)	3.40	1–2 *	3.51	1–2 *	3.00	1–2	3.04	1–2	3.55	1–2 *
	average/poor (3)	3.06	2–3 *	3.12	2–3 *	2.75	2–3 *	2.69	2–3 *	3.15	2–3 *
			1–3 *		1–3 *		1–3 *		1–3 *		1–3 *
Number of pregnancies	first pregnancy (1)	3.51	0.003	3.63	0.022	3.01	0.135 (-)	3.15	0.001	3.60	0.077 (-)
	second pregnancy (2)	3.34	1–2 *	3.47	1–2	2.88		2.94	1–2 *	3.54	
	third or subsequent pregnancy (3)	3.30	2–3	3.43	2–3	2.96		2.83	2–3	3.43	
			1–3 *		1–3 *				1–3 *		
Treatment	diabetic diet + exercise	3.41	0.582	3.56	0.083	2.99	0.039	3.02	0.213	3.55	0.336
	diabetic diet + insulin	3.37		3.45		2.87		2.93		3.49	

* adjusted significance.

**Table 6:** Acceptance of Illness Scale (AIS) and Generalized Self-Efficacy Scale (GSES) scores in the hyperglycemic pregnant women studied.

	M	SD	Me
AIS	31.37	6.38	32.00
GSES	31.58	4.60	30.00

M—mean; SD—standard deviation; Me—median.

**Table 7:** Regression analysis for WHOQOL-BREF domains and BSSS, AIS, and GSES scores in the hyperglycemic pregnant women studied.

		WHOQOL-BREF Domains															
Predictor		Physical Health F = 14.380 *p* < 0.001				Psychological F = 47.962 *p* < 0.001				Social Relationships F = 25.672 *p* < 0.001				Environment F = 38.653 *p* < 0.001			
		*B*	*β*	*t*	*p*	*B*	*β*	*t*	*p*	*B*	*β*	*t*	*p*	*B*	*β*	*t*	*p*
**BSSS Subscales**	Perceived Emotional Support	0.362	0.107	10.312	0.190	0.390	0.084	1.276	0.203	0.872	0.175	2.334	0.020	0.945	0.203	2.943	0.003
	Perceived Instrumental Support	0.343	0.117	10.453	0.147	0.937	0.232	3.584	<0.001	0.522	0.121	1.634	0.103	0.660	0.164	2.405	0.017
	Need for Support	−0.213	−0.065	−10.027	0.305	−0.219	−0.049	−0.953	0.341	0.145	−0.030	−0.516	0.606	−0.335	−0.075	−1.393	0.165
	Support Seeking	0.067	0.025	0.380	0.704	0.139	0.039	0.719	0.473	0.275	0.072	1.163	0.246	0.145	0.040	0.715	0.475
	Actually Received Support	0.085	0.026	0.418	0.677	0.338	0.077	1.505	0.133	0.765	0.162	2.782	0.006	0.855	0.194	3.618	<0.001
AIS		0.073	0.270	40.952	<0.001	0.153	0.415	9.460	<0.001	0.085	0.215	4.284	0.000	0.079	0.214	4.635	<0.001
GSES		0.055	0.147	20.684	0.008	0.088	0.171	3.876	<0.001	0.077	0.141	2.795	0.005	0.084	0.164	3.543	<0.001

WHOQOL-BREF—The World Health Organization Quality of Life—BREF; BSSS—Berlin Social Support Scales; AIS—Acceptance of Illness Scale. GSES—Generalized Self-Efficacy Scale.
